# Non-Nucleoside Lycorine-Based Analogs as Potential DENV/ZIKV NS5 Dual Inhibitors: Structure-Based Virtual Screening and Chemoinformatic Analysis

**DOI:** 10.3390/metabo14100519

**Published:** 2024-09-26

**Authors:** Adrián Camilo Rodríguez-Ararat, Yasser Hayek-Orduz, Andrés-Felipe Vásquez, Felipe Sierra-Hurtado, María-Francisca Villegas-Torres, Paola A. Caicedo-Burbano, Luke E. K. Achenie, Andrés Fernando González Barrios

**Affiliations:** 1 Grupo Natura, Faculty of Engineering, Design, and Applied Sciences, Universidad ICESI, Cali 760031, Colombia; adrian.rodriguez1@u.icesi.edu.co (A.C.R.-A.); m.f.villegastorres@uniandes.edu.co (M.-F.V.-T.); pacaicedo@icesi.edu.co (P.A.C.-B.); 2 Grupo de Diseño de Productos y Procesos (GDPP), Department of Chemical and Food Engineering, Universidad de los Andes, Bogotá 111711, Colombia; y.hayek10@uniandes.edu.co (Y.H.-O.); af.vasquez231@uniandes.edu.co (A.-F.V.); f.sierra10@uniandes.edu.co (F.S.-H.); 3 Naturalius SAS, Bogotá 110221, Colombia; 4 Centro de Investigaciones Microbiológicas (CIMIC), Department of Biological Sciences, Universidad de los Andes, Bogotá 111711, Colombia; 5 Department of Chemical Engineering, Virginia Tech (Virginia Polytechnic Institute and State University), 298 Goodwin Hall, Blacksburg, VA 24061, USA; achenie@vt.edu

**Keywords:** DENV3, ZIKV, molecular docking, MD simulations, MM/GBSA, compound library, NS5, lycorine

## Abstract

Dengue (DENV) and Zika (ZIKV) virus continue to pose significant challenges globally due to their widespread prevalence and severe health implications. Given the absence of effective vaccines and specific therapeutics, targeting the highly conserved NS5 RNA-dependent RNA polymerase (RdRp) domain has emerged as a promising strategy. However, limited efforts have been made to develop inhibitors for this crucial target. In this study, we employed an integrated in silico approach utilizing combinatorial chemistry, docking, molecular dynamics simulations, MM/GBSA, and ADMET studies to target the allosteric N-pocket of DENV3-RdRp and ZIKV-RdRp. Using this methodology, we designed lycorine analogs with natural S-enantiomers (LYCS) and R-enantiomers (LYCR) as potential inhibitors of non-structural protein 5 (NS5) in DENV3 and ZIKV. Notably, 12 lycorine analogs displayed a robust binding free energy (<−9.00 kcal/mol), surpassing that of RdRp-ribavirin (<−7.00 kcal/mol) along with promising ADMET score predictions (<4.00), of which (LYCR728-210, LYCS728-210, LYCR728-212, LYCS505-214) displayed binding properties to both DENV3 and ZIKV targets. Our research highlights the potential of non-nucleoside lycorine-based analogs with different enantiomers that may present different or even completely opposite metabolic, toxicological, and pharmacological profiles as promising candidates for inhibiting NS5-RdRp in ZIKV and DENV3, paving the way for further exploration for the development of effective antiviral agents.

## 1. Introduction

Dengue and Zika fever represent two neglected infectious diseases (NIDs) of significant global concern [1]. These diseases are particularly prevalent in tropical and subtropical regions across Africa, the Americas, Asia, and the Pacific [2,3], and are caused by dengue (DENV) and Zika (ZIKV) viruses, both belonging to the Flaviviridae family, characterized as positive-sense single-stranded RNA (+ssRNA) viruses [4]. Their transmission occurs primarily through the bite of infected Aedes mosquitoes [5,6,7], with shared urban transmission cycles and initial clinical symptoms. However, the severity of complications and sequelae differ significantly between the two diseases. DENV infection can lead to severe bleeding, organ impairment, and plasma leakage [8,9], whereas ZIKV is associated with complications such as microcephaly, Guillain–Barré syndrome (GBS), and other congenital neurological disorders [10,11]. Despite ongoing efforts in disease surveillance, diagnosis, and prevention, specific treatment options for these diseases remain elusive [8,9]. Consequently, it is imperative to develop effective drug discovery strategies for targeted infection therapies. Urgency is heightened by the co-circulation of emerging DENV/ZIKV cases, particularly in Latin American countries, such as Brazil, Venezuela, and Colombia [6,12]. Therefore, there is an urgent need to identify chemical compounds capable of effectively blocking DENV and ZIKV replication with high efficacy, selectivity, and a favorable safety profile.

**Figure 1:**
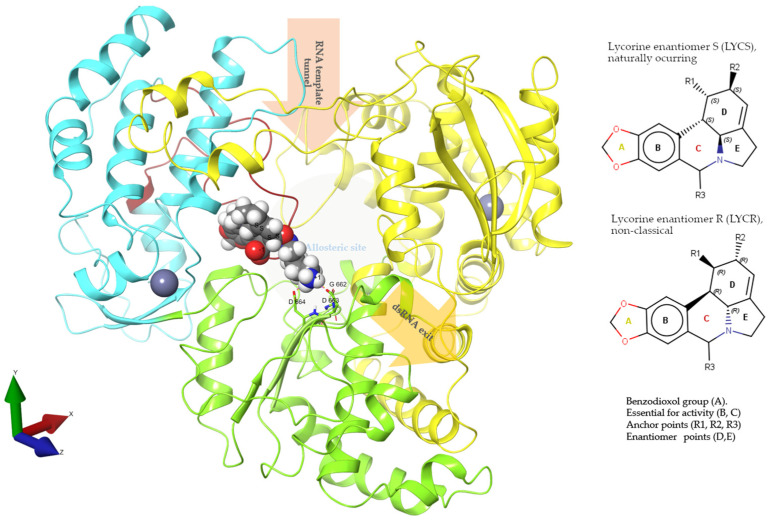
“Front view” of the overall structure of the DENV-3 RdRp (PDB code 5f3z) with lycorine analog LYCS505-214 as substrate. A ribbon diagram of the RdRp based on the report by Noble et al. (pdb entry 5F3Z) [17,18]. Interaction between LYCS505-214 (gray molecule) and the active-site residues (Asp663 and Asp664 from the GDD motif, represented as sticks), palm domain (green). The fingers and thumb domains are colored blue and yellow, respectively. The priming loop is colored red, and zinc atoms (gray). Left: Lycorine-(S) and lycorine-(R) enantiomer base structure, benzodioxol group (A), points essential for activity (B,C) [16,19]; anchor points (R1, R2, R3) and enantiomer points (D,E) used in this investigation.

The RNA-dependent RNA polymerase domain (RdRp), positioned within the C-terminal segment of the NS5 (nonstructural 5) protein, is a key target for the development of novel small-molecule drugs to combat DENV and ZIKV. This zinc metalloenzyme, found consistently among flaviviruses and notably absent in humans, plays a central role in viral genome replication [4,13]. Comprised of three subdomains—thumb (priming loop), fingers (motifs G, B, F), and palm (active site) (see Figure 1 and Figure S4)—NS5-RdRp governs RNA and NTP entry and binding, as well as the exit of the newly synthesized double-stranded RNA (dsR-NA) product. Although a handful of broad-spectrum inhibitors capable of binding and inhibiting DENV and ZIKV NS5-RdRp have been identified over the past decade [14,15], none have progressed as viable drug candidates because of concerns related to low efficacy and potential toxicity. However, recent attention has turned toward the promising potential of non-nucleoside inhibitors (NNIs), which can bind to allosteric druggable sites and induce conformational changes that affect protein function. Notably, lycorine (depicted in Figure 1, left side), a natural plant alkaloid, has shown the ability to impede RNA replication in DENV and ZIKV through weak binding to NS5-RdRp; however, a comprehensive understanding of the molecular mechanism remains elusive [16]. Given the compact size of lycorine, it serves as an optimal foundational core for the structural design and exploration of drug-sized analogs, thus enhancing its inhibitory potential against NS5-RdRp for both flaviviruses.

**Figure 2:**
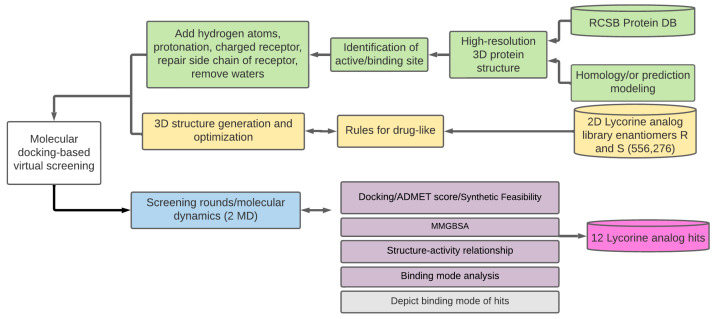
General workflow of molecular docking calculations. Target DENV3 and ZIKV 3D structure preparation and structure comparison. Lycorine analog library ligand preparation: protonation states and partial charges. Screening round by molecular docking, ADMET score, and molecular dynamics.

This research aimed to model novel non-nucleoside lycorine-based compounds with the potential to serve as dual inhibitors of DENV3 and ZIKV NS5-RdRp. We conducted a comprehensive in silico protocol integrating structure-based virtual screening and chemoinformatic analysis through combinatorial chemistry, docking, molecular dynamics simulations, MM/GBSA, and ADMET studies (Figure 2). Initially, we performed virtual enumeration of a chemical library using lycorine as the core scaffold, generating a series of analogs encompassing both the naturally occurring enantiomeric isoform, S (LYCS), and the non-classical isoform, R (LYCR), at ring D of the structure (Figure 1, left side). Subsequently, these analogs were subjected to rigorous evaluation through docking screening, molecular dynamics (MD) simulations, and MM/GBSA calculations. Additionally, the molecular descriptors and ADMET properties were estimated and compared for the complete set of designed compounds. Our analysis identified several analogs with inhibitory potential against either DENV3 or ZIKV NS5-RdRp. More significantly, certain analogs exhibited dual inhibitory potential, effectively targeting this crucial protein domain in both viral agents. Notably, analogs featuring the non-classical isoform RRRR were predicted to display more favorable interaction profiles with the target enzyme. We propose that the lycorine-based compounds assessed represent a significant breakthrough in the pursuit of broad-spectrum small-molecule drugs based on natural products for combating DENV3/ZIKV and potentially other closely related flaviviruses.

Lycorine’s traditional use in cancer research has been well established. Extracted from *Lycoris radiata*, a plant used in traditional Chinese medicine, lycorine has exhibited diverse pharmacological activities, including the induction of cancer cell apoptosis, anti-inflammatory, anti-fungal, anti-malarial, anti-tumor, and antiviral effects [20,21,22,23]. Its antiviral activity has been described in the context of RNA viruses like coronavirus, where it showed potent inhibition of replication in vitro [24,25,26]. The anti-tumor properties of lycorine were first documented in 1976, when Jimenez et al. discovered its anti-neoplastic effects [27]. Successive research confirmed its effectiveness against various cancer cell lines, as well as in inhibiting tumor growth in mouse models [28]. While lycorine’s anti-cancer properties have been extensively explored, its emerging role as an antiviral agent has gained attention, as highlighted in the introduction of this work. This shift underscores its broader therapeutic potential, and our work contributes to this evolving field by presenting lycorine analogs as viable candidates for antiviral drug development, particularly for targeting the NS5-RdRp enzyme in DENV and ZIKV.

## 2. Materials and Methods

### 2.1. Preparation of DENV3 and ZIKV RdRp NS5 Structures

The crystal structures of the DENV serotype 3 RdRp (PDB: 5F3Z) and ZIKV RdRp (PDB: 6LD5) were selected as targets for screening compounds with potential inhibitory effects. The protein structures for docking and MD simulations were prepared using Protein Preparation Wizard at a pH of 7.4, similar to cytosolic pH [29]. Crystallized water molecules, and other HETATM records were removed. Polar hydrogen atoms were added using the Epik module (Schrödinger, LCC, New York, NY, USA). Finally, restricted minimization of protein structures was performed using the OPLS3 force field with an RMSD of 0.18 Å.

### 2.2. Generation of Lycorine Analog Library

Lycorine analogs with both S and R enantiomers were synthesized to evaluate their inhibitory activity against the NS5 RNA-dependent RNA polymerase (RdRp) of Zika virus (ZIKV) and Dengue virus serotype 3 (DENV3). Lycorine-derived molecules were constructed to incorporate central carbon enantiomers by structure enumeration involving specific functional groups anchored at various points of the base structure (Figure S1) and filtered by ADMET properties analysis (Figure 2), resulting in a library containing 556,276 lycorine analogs. The use of lycorine R enantiomers against the NS5 RdRp of ZIKV and DENV3 targets provides new in silico insights into the potential inhibitory activity differences between the two enantiomers and reveals aspects of their antiviral properties. A subsequent search for structural similarity of lycorine analogs was performed in the PubChem database using the Jaccard–Tanimoto coefficient [30], with the resulting index score ranging from 0 to 1, representing the degree of intersection between pairs of elements in the compared molecules. The newly generated library, constrained within a threshold value of 0.90, formed the central structure for the anchoring points of various R groups (Figure S1), which were filtered using the Maestro program suite Schrödinger v2020-1 [11]. Moreover, a comprehensive review of the effects of prior lycorine manipulations at anchor points 1, 2, and 3 (Figure S1), as well as the nitrogen atom properties at the C-ring [31] and the stereochemistry of lycorine structure [28,31], was undertaken. Subsequently, filtered structures were selected to assess their ability to form stable complexes with the RdRp subunit of the NS5 protein of DENV3 and ZIKV. The LigPrep module (Schröding-er, LCC) facilitated the preparation of the ligand’s three-dimensional coordinates for subsequent molecular docking simulations, while the generation of ionization/tautomeric states was accomplished using the Epik module (Schrödinger, LCC) at a pH 7.4, specified chirality was retained, and no tautomer were generated. ligands were energy-minimized by applying Optimized Potential for Liquid Simulations with an OPLS-2005 force field. This comprehensive approach enhances the understanding of molecular interactions and mechanisms and also informs the design of more effective lycorine analog for future drug development processes, improving the efficacy and safety as potential antiviral agents.

### 2.3. Molecular Docking Screening

All docking calculations were carried out using the XP scoring function in Glide [32,33]. Emphasis was placed on investigating the allosteric N-pocket site located proximal to the active site of the RNA-dependent RNA polymerase (RdRp) (Figure 1 and Figure S4A,C). The selection of this docking site was justified by the mechanism of action of RNA polymerases, structure conservation, and previous studies that indicated the N-pocket of the NS5 RdRp as a key allosteric binding site for inhibiting polymerase activity in flaviviruses, which involves two aspartic acids that bind and position two metal ions, catalyzing the nucleotide transfer [17,34]. In the Zika (ZIKV) NS5 RdRp, these correspond to aspartates 535 and 665 [15], while in DENV3, they are aspartates 663 and 664 [35] (Figure 1 and Figure S4D). The active site of the ZIKV and DENV3 NS5 RdRp, as with other flavivirus RdRps, is located at the intersection of two tunnels. One tunnel, formed by the interfaces of the fingers and thumb domain, coordinates the single-strand RNA, while the second tunnel coordinates the nascent double-strand RNA (Figure 1 and Figure S4A). The priming loop, present in both DENV3 and ZIKV NS5 RdRp, is responsible for the allosteric positioning of the 3′ terminus of the RNA template at the active site [18,35].

For docking, the grid box dimensions were set to 28 × 28 × 28 Å for both DENV3 and ZIKV RdRp. The box for ZIKV-RdRp was centered around the co-crystallized ligand G80 [15] (coordinates: x = 76.1 Å, y = −2.3 Å, z = 15.4 Å), and the grid center for DENV-RdRp was positioned in the PC-79-SH52 [17] binding region (coordinates: x = 18 Å, y = 61 Å, z = 10.2 Å). A total of 556,276 compounds, including lycorine-(R) and lycorine-(S) analogs, were subjected to docking within the specified binding regions of ZIKV-RdRp and DENV3-RdRp, respectively. Compounds were ranked based on their calculated XP Glide scores (ΔG XP Glide scores). Promising candidates from the virtual screening that exhibited robust binding affinities (XP Glide score lower than −9.0 kcal/mol) for both DENV and ZIKV RdRps were selected for further analysis. To ensure the reliability of the molecular docking procedure, a validation process validated the results. This involved re-docking co-crystallized ligands from the literature (PC-79-SH52 and G8O_A_903) into their respective binding sites (PDB IDs 5F3Z and 6LD5). The validation was confirmed by comparing the Root Mean Square Deviation (RMSD) values between the co-crystallized ligand poses and the predicted poses, ensuring the reproducibility and accuracy of the docking protocol (refer to Supplementary Figure S3).

### 2.4. Molecular Dynamics Simulations

Selected ligand poses from the molecular docking process were extracted by binding affinity scoring and underwent Quantum Mechanics (QM) electronic energy minimization utilizing the RHF/6-31G(d) method. The minimized geometries were further employed for QM-restrained electrostatic potential calculations (RESP) utilizing the Merz–Singh–Kollman scheme (MK). QM calculations were executed in the gas phase by employing singlet multiplicity and Gaussian 16. The resulting RESP charges were then incorporated for ligand parameterization using the General Amber Force Field (GAFF) through Amber-Tools20. Given the presence of zinc atoms, the force field parameters for these metal ions and coordinating residues were established using the Metal Center Parameter Builder (MCPB) tool within AmberTools20. The bond and angle force constants were determined using the Seminario and Chg-ModB methods. QM calculations were performed using Gaussian 16. Parametrization of the protein atoms was carried out using the ff14SB force field, while the ligand atoms were parameterized using the GAFF force field. Additionally, the acpype.py script was utilized to convert files into Gromacs format [36].

The protein–ligand complexes were solvated within a cubic box (1.5 nm) with water molecules parameterized using the TIP3P model. To neutralize the system charge, sodium and chlorine ions were introduced at concentration of 0.15 M NaCl. The systems underwent minimization utilizing the steepest descent algorithm with a 0.01 nm step. Subsequent heating was conducted using a Berendsen thermostat, maintaining a temperature of 310 K over a simulation time of 100 ps. The initial random atomic velocities were assigned according to the Maxwell–Boltzmann distribution at 310 K. A system equilibration phase was then implemented, spanning a simulation time of 100 ps, using a Berendsen thermostat and barostat. During heating and equilibration, a harmonic potential with constant k = 1000 kJ/(mol nm^2^) was applied to restrain the backbone and ligand atoms. The Particle Mesh Ewald (PME) method was employed to address long-range electrostatic interactions, while all bonds involving hydrogen atoms were constrained using a Fourier spacing of 0.125 and a 1 nm cutoff. The entire simulation process, including heating, equilibration, and production, was performed with a time step of 2.0 fs. The production run of the systems entailed a 500 ns simulation time, utilizing the Berendsen thermostat and Parrinello–Rahman barostat. All molecular dynamics (MD) simulations were replicated, with snapshots saved every 0.1 ns and extracted from the MD trajectory. All simulations were executed using the resources provided by Virginia Tech’s Advanced Research Computing (ARC) Cluster with Nvidia T4 GPUs and MAGNUS HPC from the Universidad de Los Andes with Nvidia Tesla K40c GPUs.

Molecular dynamics (MD) simulations were evaluated with the GROMACS analysis suite [37], to assess the conformational flexibility, solvent exposure, structural compactness, and dynamic interactions of the DENV and ZIKV NS5 RdRp proteins bound to LYCR and LYCS ligands. Principal component analysis (PCA) was performed, where the covariance matrix of the Cα atomic positions was computed via gmx covar, followed by diagonalization to obtain eigenvectors and eigenvalues. The first two principal components (PC1 and PC2) were projected using gmx anaeig to assess the collective motions of the protein–ligand complexes. Solvent-accessible surface area (SASA) was calculated using the gmx sasa tool, to measure solvent exposure over the trajectory. The radius of gyration (Rg) was computed with gmx gyrate to monitor protein compactness, and hydrogen bond (Hbond) analysis was performed using gmx hbond, with hydrogen bonds defined by a donor–acceptor distance <3.5 Å and angle >150°. All results were analyzed and plotted to assess the stability and interactions of the protein–ligand complexes across the 500 ns timescale.

### 2.5. MM/GBSA Free Energy Calculations

Prime module of the Maestro program suite Schrödinger v2020-1 [11] was used to calculate the energy of the optimized complex, free receptors, and ligands. Binding free energy change calculations were performed using molecular mechanics generalized Born surface area (MM/GBSA) calculations using the formula ΔG(bind) = ΔG(solv) + ΔE(MM) + ΔG(SA), where ΔGsolv is the difference in the GBSA solvation energy of the RdRp/lycorine complex and the sum of the solvation energies for the unliganded complex, ΔEMM is the difference in the minimized energies between the RdRp/lycorine complex and the sum of the energies of the unliganded complex, and ΔGSA is the difference in the surface area energies of the complex and the sum of the surface area energies of the unliganded RdRp/lycorine complex. The RdRp–ligand complexes were minimized using local optimization features in Prime Wizard of the Maestro program suite Schrödinger v2020-1 [11]. The OPLS-2005 force field was used to determine the binding energy (ΔG-bind) of each ligand, and ligand strain energy was calculated by placing the ligand in a solution autogenerated using the VSGB 2.0 suit.

### 2.6. Chemoinformatic Analysis

The physicochemical properties of the ligand structures were explored to assess their developmental potential. The canonical Simplified Molecular Input Line Entry System (SMILES) of selected lycorine analogs, including lycorine and rivabirin, were subjected to a comprehensive screening for absorption, distribution, metabolism, excretion, and toxicity using the ADMET predictor (version 10.0.0.11, Simulations Plus, Lancaster, CA, USA) and replicated with SwissADME [38]. ADMET predictor program produced an ADMET risk score, considering absorption risk, CYP risk, and TOX risk, representing the chance of general toxicity, hepatotoxicity, mutagenicity, and adherence to Lipinski’s rules of five [39]. The cumulative effect of these aspects was measured on a scale from 0 (minimal risk) to 8 (substantial risk). An ADMET risk value of ≤4 indicates an acceptable risk level for the drug. The synthetic accessibility parameter (Synth Diff) of lycorine analogs was evaluated by the ADMET predictor, scoring the synthesis complexity of a compound at a rating from 1 (straightforward synthesis) to 10 (challenging synthesis). The Synth Diff evaluation is based on molecular fragments, their frequency, and an assessment of the fragments’ complexity, considering factors like heavy atoms, macrocycles, stereocenters, spirocenters, and bridges.

### 2.7. Activity Cliff Detection and Matched Molecular Pair Analysis

Using the ADMET predictor, we employed an automated matched molecular pair analysis (MMPA) method to identify minor structural transformations or matched molecular pairs (MMPs) [40] across each lycorine analog in the dataset. For this analysis, we set parameters to allow a maximum of 40 unmatched atoms and bonds, Tanimoto similarity thresholds of 0.50, and a maximum of one site for structural changes. The resulting data on “Molecular Pairs” were used to detect subtle structural modifications that led to significant impacts on molecular properties, known as “Activity Cliffs” (ACs) [41]. To establish a framework scaffold, we utilized the Maximum Common Substructure (MCS) method, configuring rules to support a maximum atom count of 64, a maximum ring count of 32, and the exclusion of the smallest ring systems. Distribution plots were generated to visualize the changes in values across all properties. Additionally, keys were generated using Extended Connectivity Fingerprints (ECFPs) with a minimum path length of 1 and a maximum path length of 3 [42]. This comprehensive approach allowed for the identification of critical structural changes that influence the activity of lycorine analogs, providing valuable insights for further optimization and development of these compounds as potential antiviral agents.

## 3. Results

### 3.1. Generation of Lycorine Analogs Library

The library of lycorine analogs was generated by modifying specific anchor points on the lycorine scaffold. The enumeration of scaffold anchor points resulted in 798 modifications at R1, 797 at R2, and 200 at R3 (Figure S1). The ADMET Risk scores of these analogs averaged 3.2 ± 1.9, indicating an acceptable risk level. The average molecular weight of the analogs was 471.58 ± 51.69 g/mol, compared to the natural lycorine’s molecular weight of 287.31 g/mol (Figure S2). These modifications at the anchor points led to improved affinity while maintaining a low ADMET risk [4]. Library generation demonstrated enhanced activity through molecular docking studies, with acceptable ADMET properties and other endpoints achieved through minor structural changes. The resulting library provided valuable insights into structure–activity relationships (SARs), revealing structural determinants critical for biological properties, such as target-specific activity [5,6,7,8,9].

### 3.2. Molecular Docking and MMGBSA

**Figure 3:**
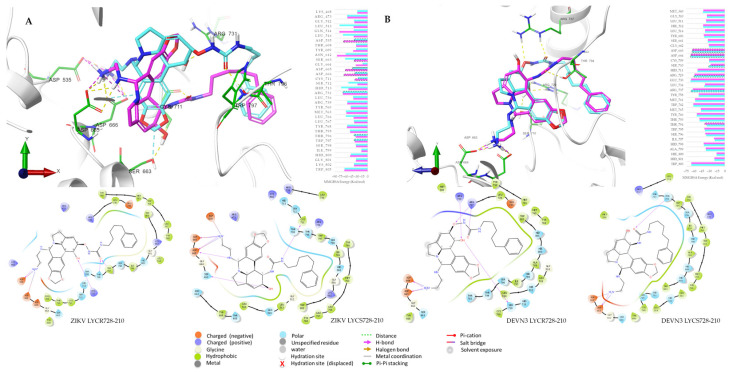
Molecular docking results: close-up view of dual lycorine analog 728-210 enantiomers R (cyan) and S (pink) at the RdRp binding site N-pocket. Interacting residues are shown in green sticks, with hydrogen bonds (yellow), π-π stacking (cyan), salt bridges (pink), and MMGBSA binding free energy profiles, highlighting the interacting amino acid residues (dashes); DENV3 (**A**) and ZIKV (**B**) RdRp.

Molecular docking and MM/GBSA analysis of the filtered lycorine analogs were carried out to evaluate their potential as inhibitors against DENV3 and ZIKV. Twelve analogs were selected based on their docking scores, binding free energies, and affinities for the NS5 RdRp enzyme (Supplementary Figures S5–S8). The docking studies revealed that the selected lycorine analogs bind effectively to the allosteric N-pocket of the NS5 RdRp enzyme. Specifically, the R3 anchor point formed hydrogen-bonding interactions with key residues at the active site such as ASP664, ASP663 and priming loop R794 in DENV3. Furthermore, the oxydiazole ring on the B ring of lycorine displayed π-π stacking interactions with HID711 in DENV3, and π-cation interactions with ARG731 in ZIKV, with binding affinities of Δ53.90 kcal/mol and Δ75.75 kcal/mol, respectively (Figure 2). Dual and single compounds exhibited two or three hydrogen bonds with active site residues and one π-π stacking interaction with HID711, showing a binding affinity of 40.12 kcal/mol (Figure 3). Docking score, binding free energy, and binding affinities of the analogs with the RNA polymerase on docking are also tabulated in Table 1. Glide XP energy delta was used to find the binding affinities of the docked hit compounds with the viral NS5 RdRp enzyme [34]. The LYCS analogs in general demonstrated lower binding affinities compared to LYCR analogs, but all compounds were found to bind within the same pocket of the protein structure, exhibiting better theoretical binding affinities than natural lycorine and with top-ranked poses. These results highlight the potential of these lycorine R enantiomer analogs as promising candidates for further studies.

Further validation of the docking results was achieved through molecular dynamics (MD) simulations, including a replica (Supplementary data: MD replica and MD PCA), performed on the selected protein–ligand complexes. The MM/GBSA analysis provided a detailed thermodynamic description of residue contributions to the binding free energy, decomposing the enthalpy value (ΔG_total, GB) at a per-residue level (Figure 3). This comprehensive analysis enhances our understanding of the interaction dynamics and stability of the lycorine analogs within the NS5 RdRp binding site.

### 3.3. Chemoinformatic Analysis

The molecular weights (MWs) ranged from 244.20 (Rivabirin) to 603.77 (LYCR727-112), and the number of heavy atoms ranged from 17 (Rivabirin) to 44 (LYCR727-112), with higher counts associated with a logical increment in the complexity of lycorine analogs. The number of hydrogen bond acceptors ranged from 5 (Lycorine) to 8 (LYCS214-510), while the number of hydrogen bond donors ranged from 1 (LYCR66-506) to 8 (LYCS214-510). Rotatable bonds varied significantly, with simpler structures like lycorine having none and more flexible molecules like LYCR728-210 having up to 14. Log P values (iLOGP [43], XLOGP3 [44], WLOGP) denoted lipophilicity, with LYCR294-114 showing the highest consensus Log P (6.00), suggesting significant lipophilicity. In contrast, Rivabirin had much lower values, reflecting its hydrophilic nature. Solubility predictions (ESOL Log S, Ali Log S, Silicos-IT LogSw) indicated that lycorine analogs like LYCR66-506 and LYCS505-214 were very soluble, whereas LYCR294-114 and LYCR727-112 were poorly soluble. ADMET risk scores were generated for absorption, distribution, metabolism, excretion, and toxicity. LYCS505-214 and LYCR66-506 had ADMET risk values (≤ 4), indicating their potential suitability for drug development. Specific risks included absorption, CYP enzyme inhibition, and general toxicity. LYCS505-214 and LYCR66-506 demonstrated favorable profiles with minimal predicted risks. The synthetic accessibility scores ranged from 4.20 (Lycorine) to 6.57 (LYCR727-112), indicating varying levels of complexity in synthesis. Lower scores suggest simpler and more feasible synthetic routes. Lipinski, Ghose, Veber, Egan, and Muegge rule violations [38] were minimal (Table 1), indicating good drug-likeness for most analogs. LYCR294-114 and LYCR727-112 are exceptions with higher synthetic difficulties (Table 2). Bioavailability scores of 0.55 for all compounds indicate moderate the likelihood of oral bioavailability. Most exhibited high GI absorption, critical for oral drug administration. None were predicted to cross the blood–brain barrier (BBB), reducing the likelihood of central nervous system (CNS) side effects. Inhibition of cytochrome P450 enzymes (CYP1A2, CYP2C19, CYP2C9, CYP2D6, CYP3A4) was minimal across the analogs (Table 2), suggesting low potential for drug–drug interactions. Several were identified as permeability glycoprotein (Pgp) substrates, which could affect drug distribution and clearance. PAINS (Pan-Assay Interference Compounds) [45] and Brenk alerts [46] were minimal, suggesting low risk of false positives in biological assays. The comprehensive chemoinformatic analysis revealed that several lycorine analogs and co-crystallized ligands demonstrated favorable physicochemical properties, ADMET profiles, and synthetic accessibility, making them promising candidates for further in vitro investigation as antiviral agents. Notably, analogs like LYCR728-210 and LYCS505-214 stood out due to their balanced profiles of solubility, lipophilicity, and synthetic feasibility and their potential capacity to target ZIKV and DENV3 NS5 RdRp. Further experimental validation and optimization were warranted to fully assess their therapeutic potential.

### 3.4. GI (Gastrointestinal System), BBB (Blood–Brain Barrier), CYP (Cytochrome P)

**Scheme 1:**
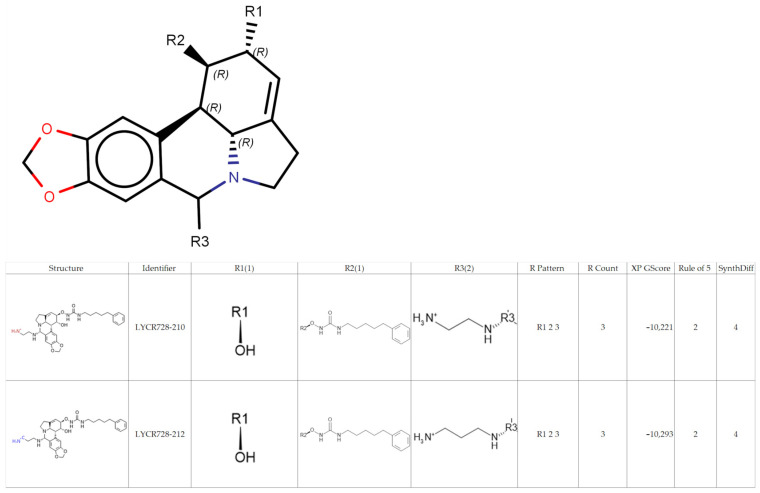
Chemical structures and activity data for Lycorine analogs LYCR728-210 and LYCR728-212. The top image represents the core structure of the Lycorine scaffold with labeled functional groups R1, R2, and R3. The table below shows specific modifications of the analogs, including the R1(1), R2(1), and R3(2) substituents, along with their corresponding XP GScore, rule of 5 compliance, and synthetic difficulty (SynthDiff) scores. Notably, LYCR728-210 and LYCR728-212 differ slightly in their R3(2) substituents, resulting in distinct XP GScores, highlighting key differences in their predicted biological activity.

Activity cliff analysis highlighted the differences between the various pairs of lycorine analogs, focusing on their structural mismatches, similarity scores, and changes in key properties (Scheme 1). The number of mismatches and distinct molecular sites where compounds differed significantly across pairs ranged from four mismatches (for highly similar pairs) to twenty-two mismatches (for more distinct pairs). Lycorine analogs LYCS728-210 and LYCR728-212 displayed high similarity scores of 0.955 and minimal changes in XP Gscore (−0.071), while LYCR66-506 and LYCS510-214 displayed an average similarity of 0.763 but the highest change in XP GScore in favor of the second analog (0.728). These changes in the XP GScore reflect the differences in binding affinity between the pairs related to the effect of the R groups added. Changes in the rule of five violations were minimal for most pairs, indicating that most analogs maintained drug-like properties. Pairs with high similarity scores and minimal changes in key properties (LYC728-210 and LYC728-212) may offer insight into minor structural modifications that can enhance activity or reduce toxicity.

SynthDiff: synthetic differential ADMET simulation plus; XP Gscore: extra-precision glide docking score.

### 3.5. Binding Pose Analysis of Lycorine

Ligand–protein interactions of DENV3-RdRp and ZIKV-RdRp are situated within the thumb domain in analogous positions (Figure 3, Supplementary Figures S5–S8). Interestingly, LYCR728-210, LYCR728-212, LYCS505-214, and LYCS728-210 were anchored by the N-pocket and priming loop in the DENV3 RdRps and ZIKV RdRps, despite residue differences, suggesting a structural tendency of the positive charge of the lycorine scaffold to be cavity-guided by the superficial polarity of the palm domain. The active site and Motif E surfaces at the palm domain are essential for the initiation of RNA replication. In the DENV3-RdRp/LYCR294-114 complex, the priming loop residues Glu 733, Arg 737, Gln 802, Trp 803 and with ZIKV-RdRp the residues Arg 731, Glu 735 made the main contribution to the total binding energy of the interaction of LYCR66-506 at N-pocket. This interaction has the potential to block RdRp activity by a potential retraction of the priming loop (aa785–807) from the active site during enzyme elongation by changing the N-pocket’s conformation, reducing the binding affinity of the RdRp for the ligands [13,15]. This structural change also has the potential to obstruct substrate access by affecting the priming loop and keep the RdRp in a “closed” state that may prevent the polymer from shifting from the initiation to the elongation stage of replication [15,47,48]. Similarly, LYCR66-506 and LYCS505-212 also interacted with highly conserved residues (GDD) in the N-pocket of ZIKV RdRp (Supplementary Figures S5–S8). Our study indicates that, despite the sequence differences between the DENV3 and ZIKV RdRps, their common allosteric sites share similar geometric and surface properties. Lycorine derivatives may be effective against other viral polymerases with similar allosteric sites. Natural products from plants and in silico derivations are promising strategies for the development of novel antiviral therapeutic agents. These developments will be great improvements in terms of time and cost. To identify DENV3 and ZIKV RdRp inhibitors using in silico derivation, we derived lycorine structures and screened them in silico. Interestingly, the findings indicate for the first time that lycorine analogs may have broad-spectrum antiviral potential. They inhibit DENV3 and ZIKV replication by targeting viral RdRps.

The derivatization of lycorine produced numerous molecules, most of which were not synthetically feasible, as is usually the case in this type of approach [49]. Reducing the synthesis feasibility space using synthetic accessibility (SA) parameters [50] allowed for the final determination of a set of hit molecules. It was considered that lycorine in its natural conformation fits within the cases presented in the literature, in which the compounds to be validated initially exist, and it was taken into account that the SA scoring methods that use the complexity method are susceptible to very optimistic evaluations, especially when starting from chemical structures that are made de novo or derived, which reduces the guarantee that these structures would be feasible, even if the SA score was positive. In our case, the compounds that were screened for validation from natural lycorine offered acceptable synthesis probability (SA), meaning that chemical synthesis should, in principle, be feasible despite possible complications [49]. The joint efforts to obtain molecules derived from lycorine have not been fruitful to date, as suggested in the synthesis feasibility evaluations conducted with our team. Among the possible factors discussed were the difficulty of some reactions, steric hindrances, and experimentally risky reactions, among others, which have been discussed in other studies with similar approaches [49].

### 3.6. Molecular Dynamics Simulation

**Figure 4:**
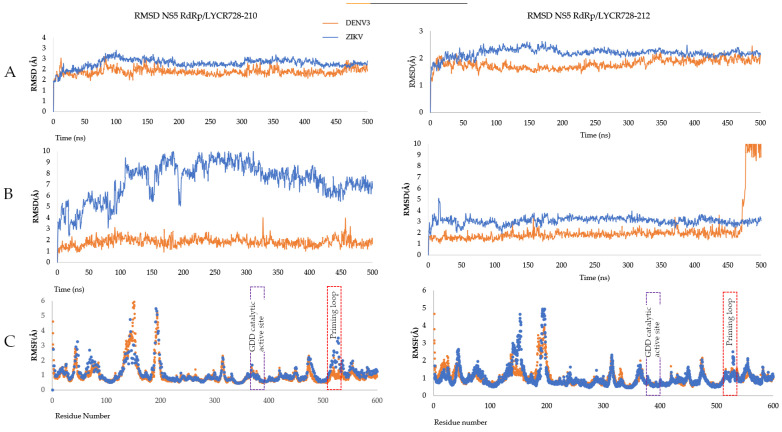
MD simulation results at a 500 ns timescale for LYCR728-210 and LYCR728-212 complexes with NS5 RdRp. (**A**) RMSD of NS5 RdRp complexes with LYCR728-210 (left) and LYCR728-212 (right). (**B**) RMSD of ligands LYCR728-210 (left) and LYCR728-212 (right) during the simulation. (**C**) RMSF of residues in NS5 RdRp in complex with LYCR728-210 (left) and LYCR728-212 (right). The orange line represents DENV3, and the blue line represents ZIKV. The highlighted regions in (**C**) correspond to the GDD catalytic active site (purple box) and the priming loop (red box), showing significant fluctuations.

The RMSD was calculated for all complexes for a 500 ns trajectory (Figures S9 and S10). The RMSD value of the NS5 RdRp/LYCR728-210 complex showed a steady pattern for ZIKV and DENV3. The RMSF plot (Figure 4) was calculated for a 500 ns simulation for all the complexes showed a ~0.5 Å deviation between residues corresponding to the palm and finger subdomains which participated in the ligand binding, in particular the priming loop and the active site (Figures S5–S8).

**Figure 5:**
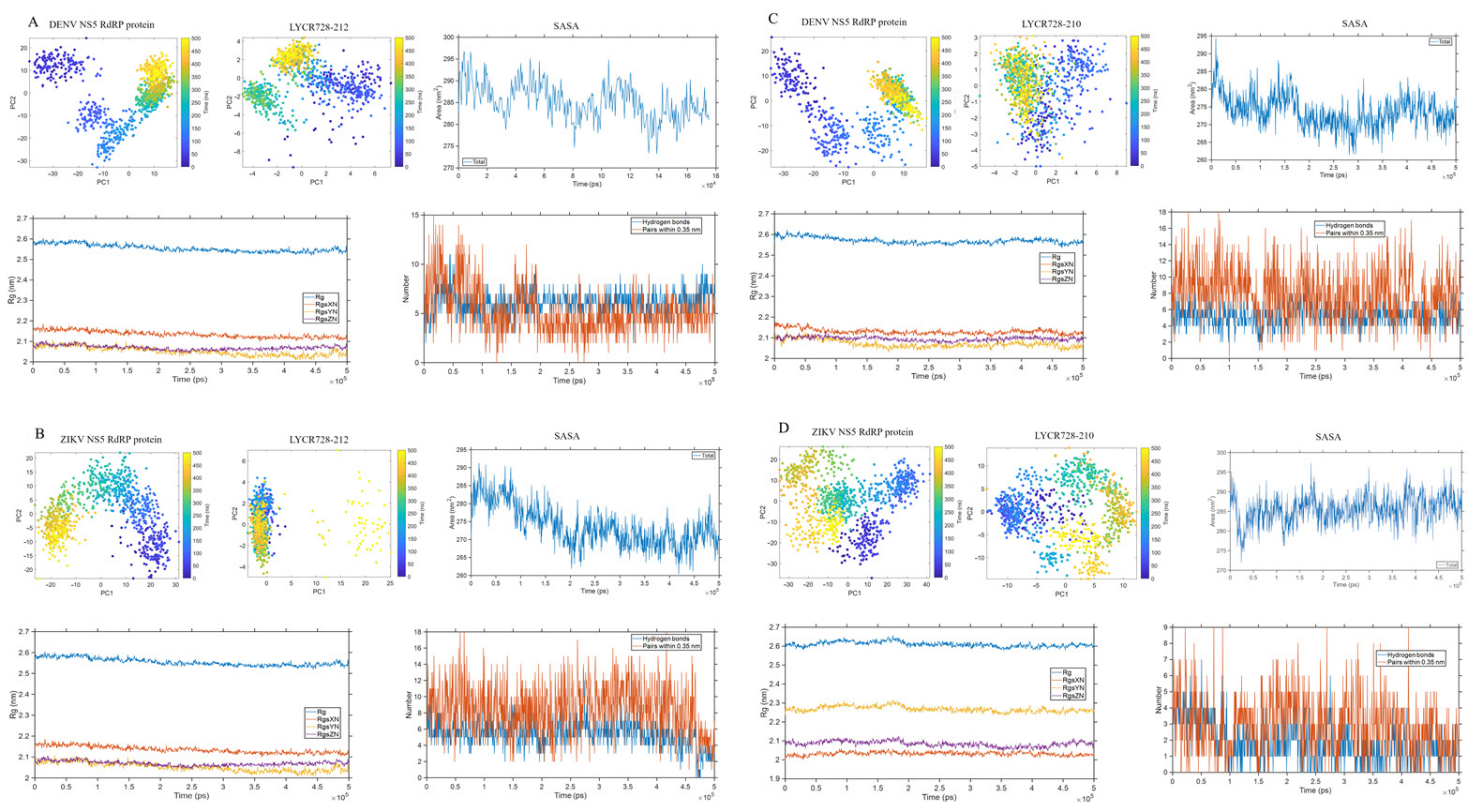
Comparative MD simulation results at 500 ns timescale of DENV and ZIKV NS5 RdRP proteins in complex with LYCR728-212 and LYCR728-210 ligands. (**A**) PCA, SASA, radius of gyration, and hydrogen bond analysis of DENV NS5 RdRP protein bound to LYCR728-212. (**B**) Similar analysis for ZIKV NS5 RdRP protein bound to LYCR728-212. (**C**) Analysis of DENV NS5 RdRP protein with LYCR728-210. (**D**) Corresponding analysis for ZIKV NS5 RdRP protein with LYCR728-210. PCA plots show protein flexibility across PC1 and PC2 with time progression (blue to yellow). SASA plots indicate solvent exposure over time, Rg plots reflect protein compactness, and hydrogen bond plots show dynamic ligand–protein interactions across the simulation.

Principal component analysis (PCA), solvent-accessible surface area (SASA), radius of gyration (Rg), and hydrogen bond (Hbonds) analyses were performed to assess the stability and flexibility of these complexes during the simulation period. For the DENV NS5 RdRP protein bound to LYCR728-212 (Figure 5A), the PCA revealed moderate conformational flexibility, particularly along PC1, with the time progression indicating a stable structural arrangement (blue to yellow gradient). The LYCR728-212 ligand exhibited minimal conformational variation, maintaining a relatively compact spread in PCA space. The SASA of the DENV NS5 protein fluctuated over time without significant long-term trends, suggesting stable solvent exposure throughout the simulation. The radius of gyration indicated that the protein structure remained compact and stable, with minimal changes in overall size. Dynamic interactions were observed in the hydrogen bonding profile as the number of hydrogen bonds between DENV NS5 and LYCR728-212 fluctuated, indicating active and transient binding throughout the simulation.

For the ZIKV NS5 RdRP protein bound to LYCR728-212 (Figure 5B), PCA showed greater conformational flexibility compared to DENV NS5, indicating a broader range of structural variance. However, the LYCR728-212 ligand maintained similar conformational stability to that observed in the DENV complex. SASA analysis demonstrated that the solvent exposure of the ZIKV NS5 protein fluctuated but did not trend towards significant expansion or compaction. Like in the case of DENV, the radius of gyration remained stable, reflecting a maintained compact structure. Hydrogen bond analysis showed dynamic and fluctuating interactions between ZIKV NS5 and LYCR728-212, akin to the DENV complex, suggesting transient binding modes. In the DENV NS5 RdRP protein complexed with LYCR728-210 (Figure 5C), PCA again showed moderate flexibility in the protein, with the LYCR728-210 ligand displaying slightly more conformational variability than LYCR728-212. SASA trends mirrored those observed in the LYCR728-212 complex, with stable solvent exposure across the simulation. The radius of gyration confirmed that the DENV NS5 protein maintained its compactness, and hydrogen bonding analysis indicated dynamic interactions, with hydrogen bonds forming and breaking continuously throughout the simulation.

The ZIKV NS5 RdRP protein complexed with LYCR728-210 (Figure 5D) exhibited similar trends in PCA, with a wider range of conformational movement than DENV NS5, as was the case with LYCR728-212. The LYCR728-210 ligand demonstrated a comparable spread to LYCR728-212 in PCA space, with minor differences in flexibility. SASA and radius of gyration analyses indicated that the ZIKV NS5 protein maintained stable solvent exposure and structural compactness throughout the simulation. Hydrogen bonds fluctuated in number, indicating dynamic interactions between the protein and LYCR728-210 ligand. Overall, the ZIKV NS5 RdRP protein consistently demonstrated greater conformational flexibility than DENV NS5 in LYCR complexes. The ligands themselves remained relatively stable throughout the simulations (Supplementary Figures S11 and S12), with some complexes, like LYCR728-210, showing slightly more flexibility than LYCR728-212. SASA trends indicated stable solvent exposure for both DENV and ZIKV NS5 proteins, and the radius of gyration data confirmed that both proteins maintained their structural integrity. The dynamic nature of hydrogen bond formation and breaking in all complexes suggests transient and flexible binding interactions between the lycorine-based ligands and the NS5 RdRP proteins.

### 3.7. Validation of the Docking Performance and Accuracy

Validation of the docking protocol involved removing the inhibitors from their respective complexes, re-docking them, and calculating the Root Mean Square Deviation (RMSD). Specifically, the inhibitors PC-79-SH52 (PDB ID 5F3Z) [17] and G8O903 (PDB ID 6LD5) [15] were chosen as templates due to their suitable ligand-binding pockets for virtual screening. Lycorine analogs were similarly docked to the N-POCKET of DENV-RdRp, interacting with active site residues.

Superimposition of the DENV-RdRp/PC-79-SH52 and ZIKV-RdRp/G8O903 complex structures showed that both crystal structures bound to a common allosteric site. The docking parameters for GLIDE were optimized by re-docking the co-crystallized ligands PC-79-SH52 [29] and G8O903 [15] into their inhibitor-binding sites. The calculated binding energies for PC-79-SH52 and G8O903 in their respective binding sites were −5.6 and −6.2 kcal/mol, respectively, with RMSD values of 1.26 and 0.44 Å (see Supplementary Figure S3). To further assess accuracy, the interactions reproduced upon re-docking were compared to those in the native conformations of PC-79-SH52 and G8O903. For G8O903, the re-docked conformation replicated the same hydrogen bonding interactions with Arg 731 and Thr 796 of the priming loop. Similarly, the re-docked PC-79-SH52 ligand’s carboxyl group formed an ionic bond with Arg 737, consistent with its native conformation. The acceptable RMSD values, both under 2.00 Å, and the reproducibility of key interactions indicated that the docking protocol was reliable and could be used effectively for further studies [51].

## 4. Discussion

### Binding Poses Analysis of Lycorine

To understand the interaction of lycorine derivates with the DENV and ZIKV RdRp, we used a theoretical approach to predict the binding mode. Published crystal structures of the DENV and ZIKV RdRps were extracted from the Protein Data Bank and docked to our lycorine derivate library in the allosteric ligand binding region (N-Pocket) of polymerase using GLIDE XP. Structures of RdRp in complexes were selected by XP Glide score (≤9.00) and ADMET predictor score (≤4.00) and relaxed using molecule dynamic simulation for 10 ns. After that, the MM/GBSA computational method was used to calculate the contribution of amino acids to the binding free energy of lycorine hits. As shown in Figure 2 (LYCR 728-210, 728-212) and Figure S10 (LYCS 505-214, 728-210), the RMSD indicated the stability of lycorine hits in both DENV and ZIKV RdRp binding sites, which confirmed that these lycorine hits were RdRp inhibitors. The superimposition of previous reported structures showed that lycorine was bound to a common site at the N-pocket of the DENV and ZIKV RdRp. As shown by the ligand interaction analysis of the binding site (Supplementary Figures S5–S8), the residues that make the main contributions to lycorine derivative LYCS728-210 binding in the DENV3 RdRp are (TRP762, ASP663, ASP664, ARG737, and TRP803), while in ZIKV, they are (ASP665, ASP66, ARG731, and ASP535).

The main interactions are hydrogen bonds between the ligand and R group of ASP residues of the active site (Figure 3). Another important interaction is π-cation and π-π stacking to the R groups with free hydroxyl groups and the benzodioxole group at the B-ring (Figure 1), like ARG729 in DENV3 versus LYCS728-210 and ARG731 in the ZIKV NS5 RdRp in complex with LYCR211-507. Studies on the structure–activity relationship of natural lycorine revealed that the free hydroxyl groups at C-1, C-2, the intact benzodioxole group at the B-ring, the basic nitrogen, and the C3-C4 double bond are crucial for the antivirus activity of lycorine [31].

Lycorine analogs LYCS and R anchor to the priming loop of DENV3 and ZIKV (~aa790–807) at the N-pocket and interact with the polar residues (THR793, THR794, THR796, and Ser 796) and hydrophobic residues (TRP793, TRP797, and Ile 799). The priming loop is one of the special characteristics of the flavivirus RdRp and plays a key role in de novo RNA initiation. The interaction between lycorine analogs and key priming loop residues has the potential to keep the polymerase in a closed conformation and, therefore, halt viral RNA synthesis. As shown in Figure 3, Lycorine analogs bind to the equivalent allosteric site N-Pocket in the DENV and ZIKV RdRp.

The primary contributing amino acids in the DENV3 N-Pocket binding cavity were ASP663, ASP664, ARG731, and ASN612. These residues form the key interactions between the ligand and the receptor for both LYCS and LYCR analogs. Notably, there is a cationic π-interaction between ring B (Figure 1) of LYCR211-507 and the side chain of ARG731, similar to the interaction reported with RAI-13 [52].

Principal component analysis (PCA) revealed a moderate conformational flexibility for DENV and ZIKV bound to LYCR, with fluctuations primarily along the first principal component (PC1), suggesting a stable global structure despite small-scale dynamic movements. The LYCR728-212 ligand displayed limited conformational variability, maintaining a compact presence throughout the simulation, as indicated by its restricted spread in the PCA space. SASA analysis suggested that the solvent exposure of the DENV NS5 protein remained relatively stable, with no significant long-term trends in fluctuation, highlighting the consistent interaction between the protein and its solvent environment. The radius of gyration analysis showed that the protein retained a compact structure, while hydrogen bond dynamics demonstrated a transient yet consistent interaction between the protein and LYCR728-212, further suggesting active and flexible binding.

## 5. Conclusions

This research highlights the potential of lycorine-based analogs as dual inhibitors of DENV3 and ZIKV NS5 RdRp. Through a combination of cheminformatics, molecular docking, and molecular dynamics simulations, several compounds with favorable binding affinities and ADMET properties were identified. LYCR728-210 exhibited strong binding affinity to both the DENV3 and ZIKV NS5 RdRp, with a binding free energy of −9.2 kcal/mol, and LYCS505-214 demonstrated dual inhibitory activity, suggesting its potential for treating co-infections. Key interactions with the NS5 RdRp enzyme, such as hydrogen bonding with ASP664, ASP663, and R794 in DENV3 and ARG731 in ZIKV, were central to these findings. Molecular dynamics simulations further supported the stability of the protein–ligand complexes, indicating sustained inhibitory potential. However, in vitro and in vivo studies are needed to validate these computational predictions, assess compound selectivity, and evaluate pharmacokinetics. These experimental efforts will address the limitations of in silico methods and provide a clearer understanding of their therapeutic potential.

The dynamic hydrogen bond behavior and stability observed in the DENV and ZIKV NS5 RdRp complexes with LYCR728-212 and LYCR728-210 align with previous studies in antiviral drug development, reinforcing the potential of lycorine analogs as dual inhibitors. Future efforts will focus on optimizing these compounds, conducting preclinical studies, and evaluating their clinical efficacy. In vitro assays will be essential to confirm the inhibitory activity and further align molecular dynamics predictions with biological outcomes.

PCA and comparative analyses of dynamic hydrogen bond formation and the structural stability of DENV and ZIKV NS5 RdRp complexes with LYCR728-212 and LYCR728-210 (Figure 5) align with previous antiviral studies, reinforcing the hypothesis that molecular stability in simulations can predict inhibitory efficacy. Optimizing these compounds, conducting preclinical studies, and evaluating their clinical potential are crucial next steps. In vitro assays will be key in confirming the computational predictions and bridging them with biological outcomes.

## Figures and Tables

**Table 1:** Predicted drug-likeness based on the Lipinski, Ghose, Veber, Egan, and Muegge rules and synthetic accessibility of the selected compounds.

Molecule	Lipinski	Ghose	Veber	Egan	Muegge	Synthetic Accessibility
Rivabirin	0	1	1	1	0	3.89
Lycorine	0	0	0	0	0	4.2
LYCS214-507	0	1	0	0	0	4.94
LYCS505-214	0	1	0	0	1	5.04
LYCR66-506	0	0	0	0	0	5.09
LYCS510-212	0	1	0	0	0	5.18
LYCR211-507	0	1	0	0	0	5.27
LYCS510-214	0	2	1	0	0	5.31
LYCS214-510	0	3	1	0	0	5.76
LYCS728-210	1	3	1	0	0	5.87
LYCR728-210	1	3	1	1	0	6.11
LYCR728-212	1	3	1	1	0	6.24
LYCR294-114	1	3	1	0	0	6.41
LYCR727-112	1	3	1	0	1	6.57

**Table 2:** Predicted ADMET properties of the selected compounds.

Molecule	GI Absorption	BBB-Permeant	Bioavailability Score	CYP Substrate/Inhibitor				
				CYP1A2	CYP2C19	CYP2C9	CYP2D6	CYP3A4
Rivabirin	Low	No	0.55	No	No	No	No	No
Lycorine	High	No	0.55	No	No	No	Yes	No
LYCS214-507	High	No	0.55	No	No	No	No	No
LYCS505-214	High	No	0.55	No	No	No	No	No
LYCR66-506	High	No	0.55	No	No	No	No	No
LYCS510-212	High	No	0.55	No	No	No	No	No
LYCR211-507	High	No	0.55	No	No	No	No	No
LYCS510-214	High	No	0.55	No	No	No	No	No
LYCS214-510	High	No	0.55	No	No	No	No	Yes
LYCS728-210	High	No	0.55	No	No	No	No	Yes
LYCR728-210	High	No	0.55	No	No	No	No	Yes
LYCR728-212	High	No	0.55	No	No	No	No	Yes
LYCR294-114	High	No	0.55	No	No	No	No	No
LYCR727-112	High	No	0.55	No	No	No	No	Yes

## Data Availability

Supplementary Data can be found here: https://github.com/Grupo-de-Diseno-de-Productos-y-Procesos/Lycorine-analogs.git, accessed on 5 July 2024. Additional raw data will be available upon request.

## Supplementary Materials

The following supporting information can be downloaded at https://www.mdpi.com/article/10.3390/metabo14100519/s1, Figure S1: Two-dimensional structure of the natural product lycorine; Figure S2: Lycorine enumeration and ADMET property analysis; Table S1: Docking results (extra precision glide scores—XP GScore); Table S2: GLIDE/ADMET analysis of natural lycorine; Table S3: Analysis of the activity cliffs for lycorine analogs; Figure S3: Re-docking of crystallographic ligands; Figure S4: (A) ZIKV RdRp complex, (B) colored surface of electrostatic potential, (C) DENV3 and ZIKV RdRp sequence alignment; Figures S5–S8. XP glide docking pose and 2D diagram of the interaction results of lycorine enantiomers; Figures S9 and S10: Molecular dynamics simulation (MDS) results of DENV3 and ZIKV RdRp–ligand complex; Figures S11 and S12: Comparative MD simulation results at 500 ns timescale of DENV and ZIKV NS5 RdRP proteins in complex with LYCR and LYCS ligands. PCA, SASA, radius of gyration, and hydrogen bond analyses of DENV and ZIKV NS5 RdRP protein bound to LYCR/S.
