# Supplementary figures and images for: Non-Nucleoside Lycorine-Based Analogs as Potential DENV/ZIKV NS5 Dual Inhibitors: Structure-Based Virtual Screening and Chemoinformatic Analysis
Source: Metabolites. 2024 Sep 26;14(10):519. doi: 10.3390/metabo14100519 (PMC11509260; doi:10.3390/metabo14100519)

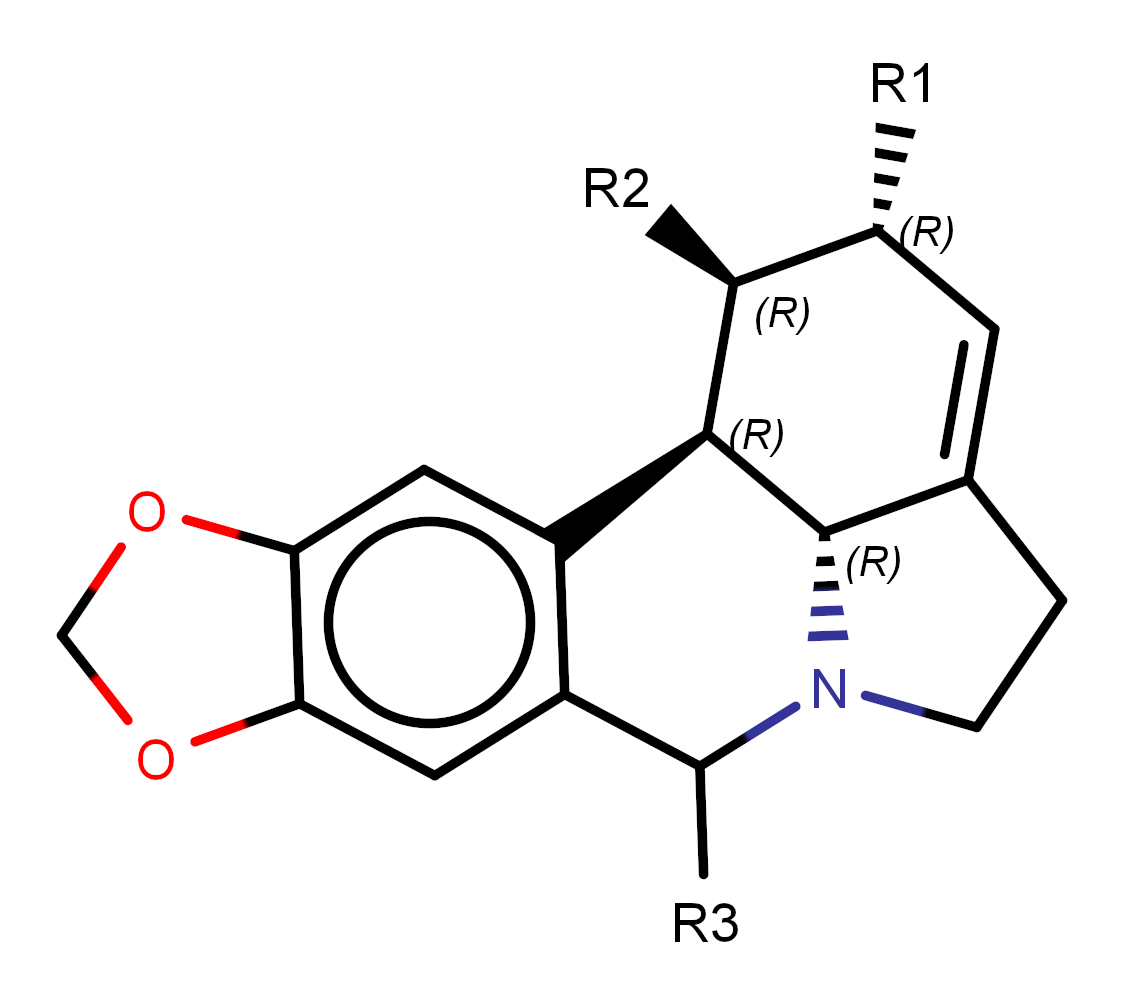

Supplement: Supplementary file 1 [file metabolites-14-00519-s001.zip › Figure S1.png]

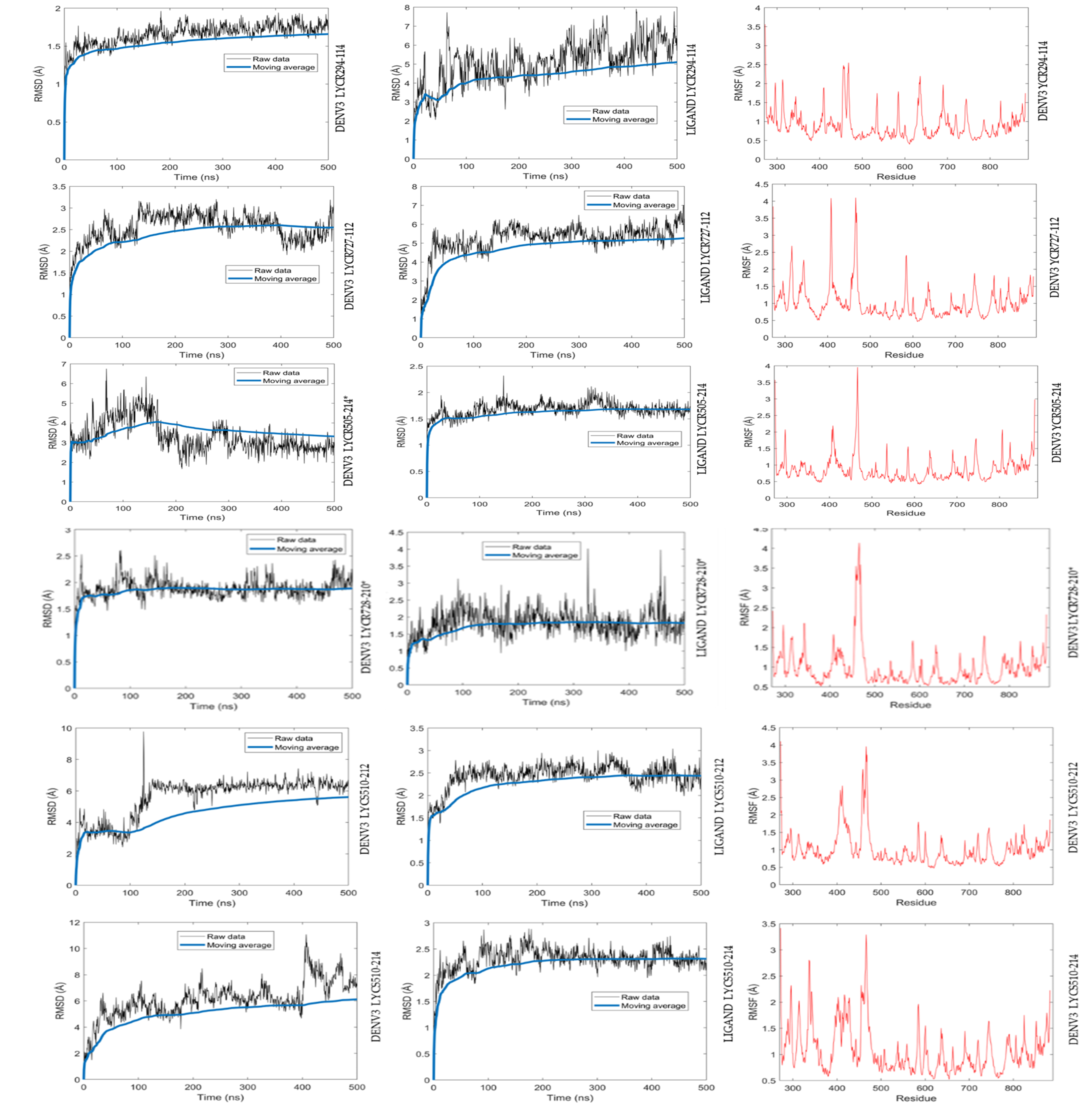

Supplement: Supplementary file 1 [file metabolites-14-00519-s001.zip › Figure S10.png]

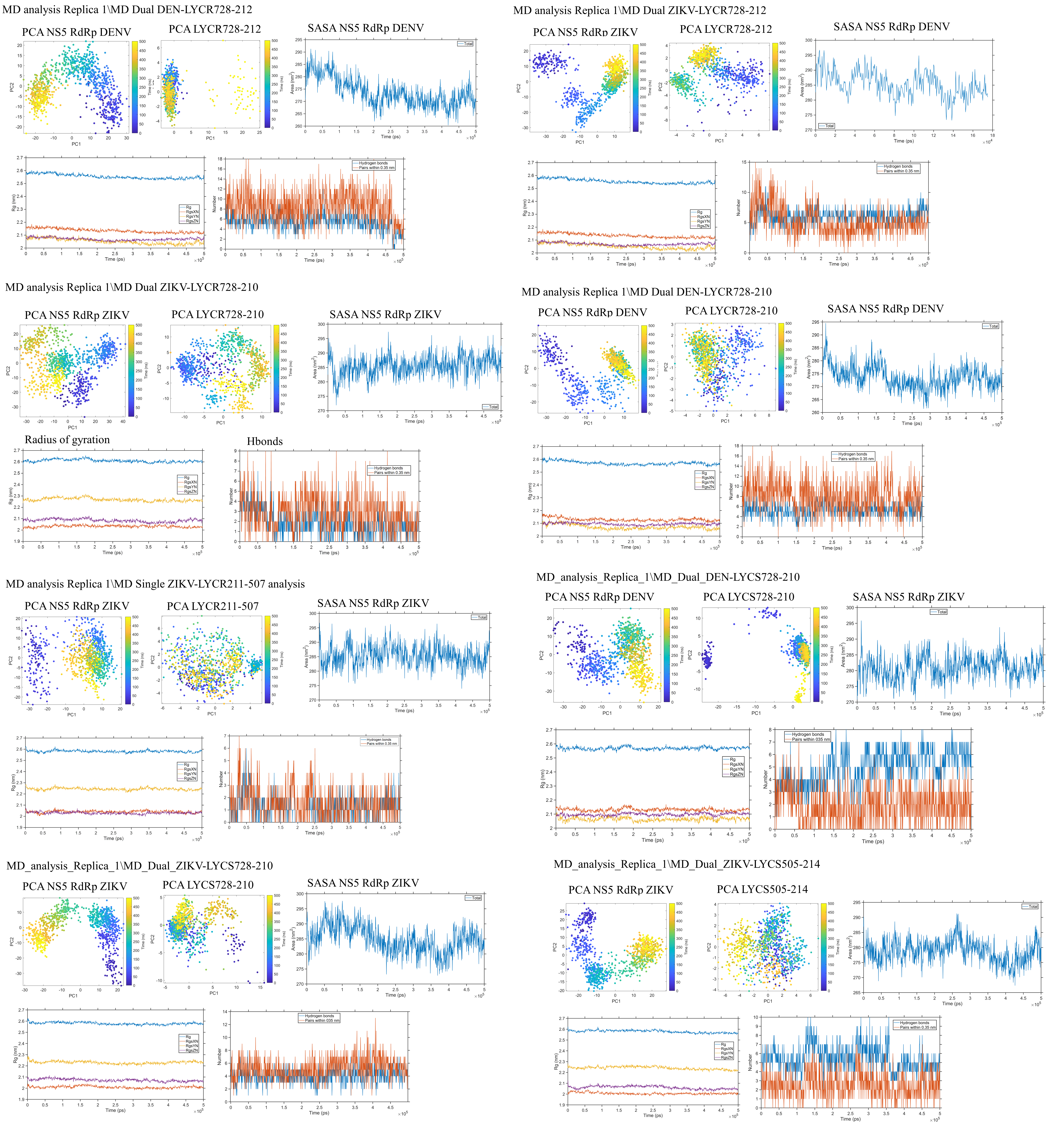

Supplement: Supplementary file 1 [file metabolites-14-00519-s001.zip › Figure S11 MD_PCA_SASA_Rg_Hbonds.png]

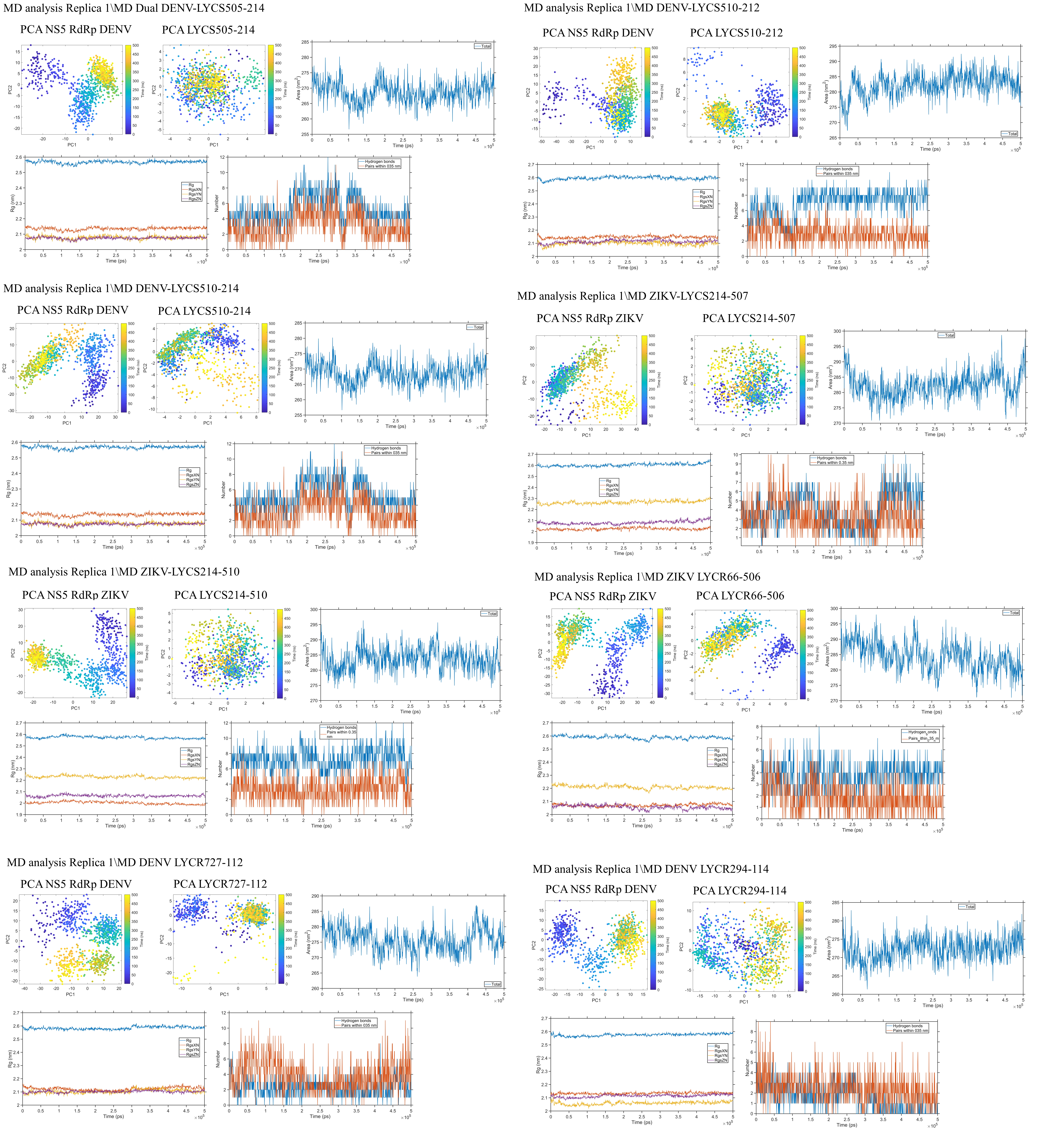

Supplement: Supplementary file 1 [file metabolites-14-00519-s001.zip › Figure S12 MD_PCA_SASA_Rg_Hbonds.png]

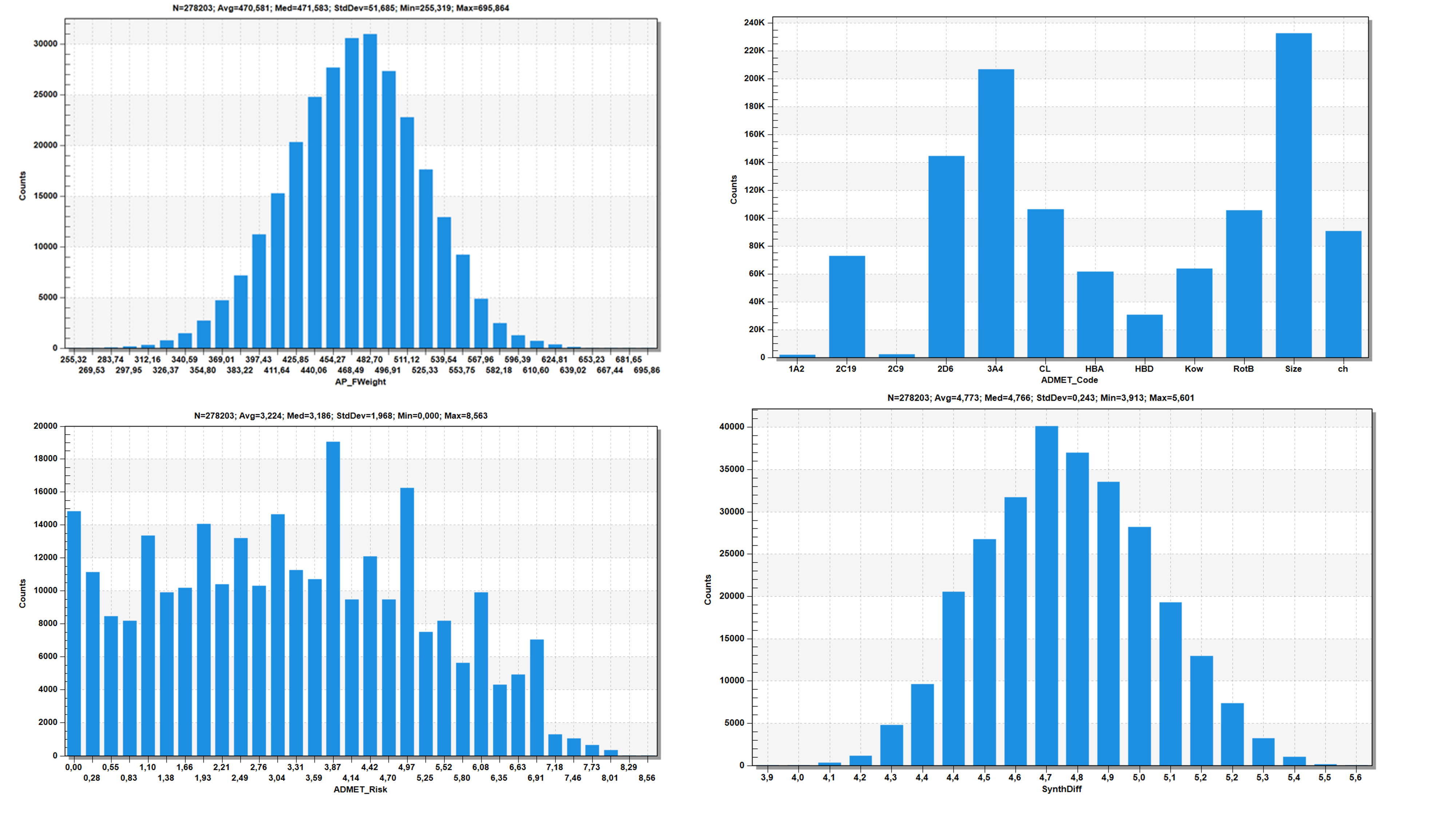

Supplement: Supplementary file 1 [file metabolites-14-00519-s001.zip › Figure S2.png]

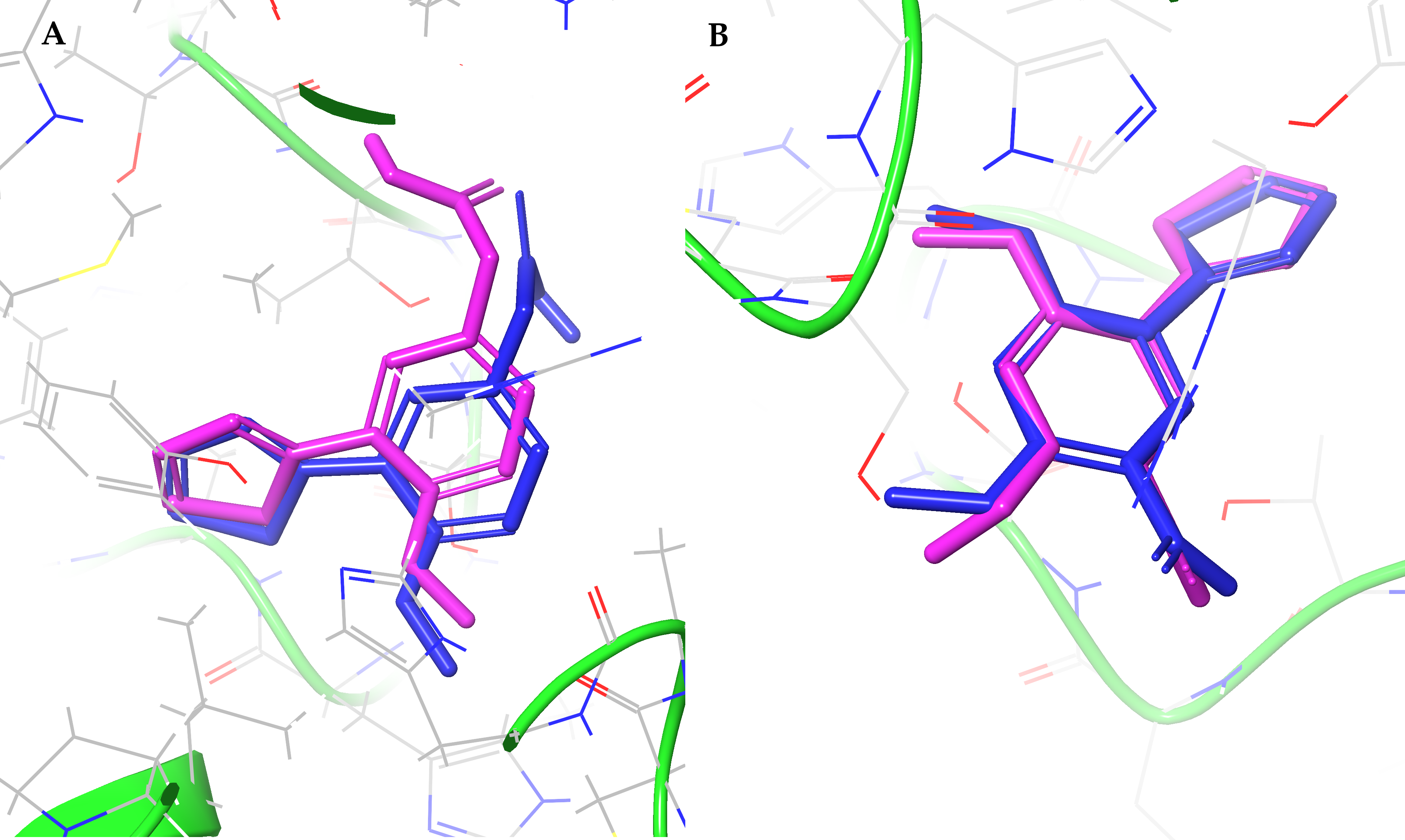

Supplement: Supplementary file 1 [file metabolites-14-00519-s001.zip › Figure S3 Redocking of crystallographic ligands.png]

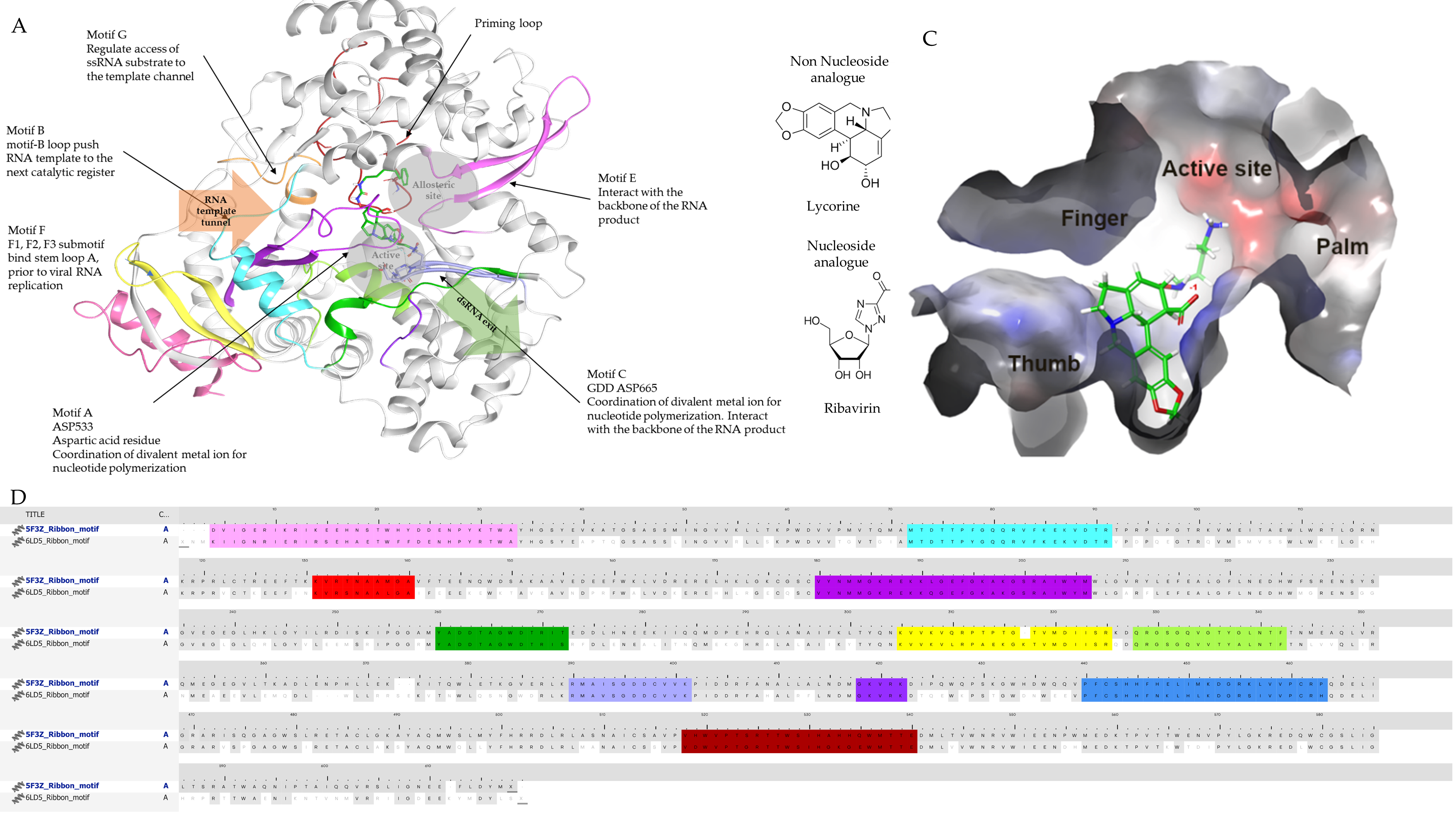

Supplement: Supplementary file 1 [file metabolites-14-00519-s001.zip › Figure S4.png]

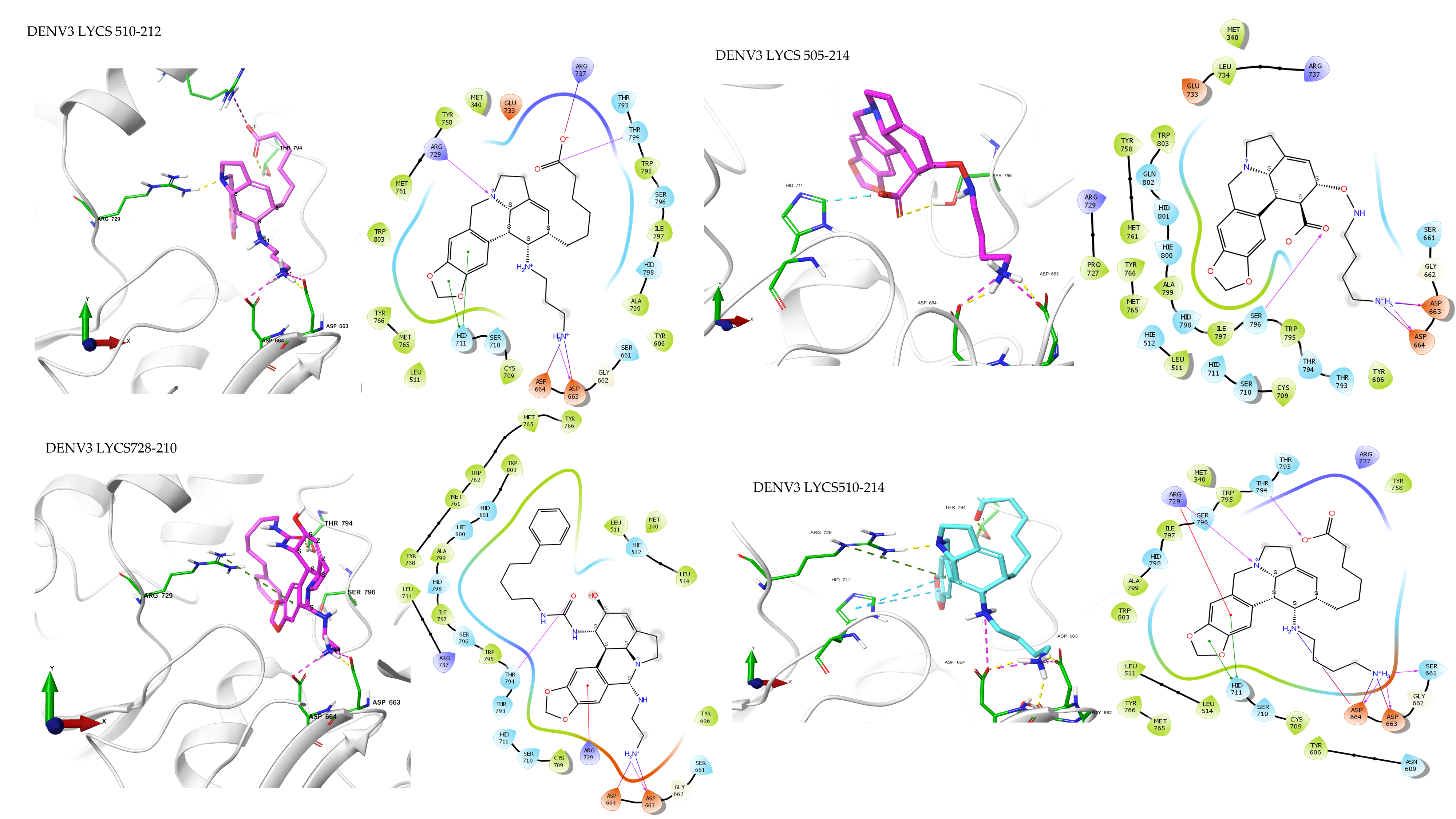

Supplement: Supplementary file 1 [file metabolites-14-00519-s001.zip › Figure S5.png]

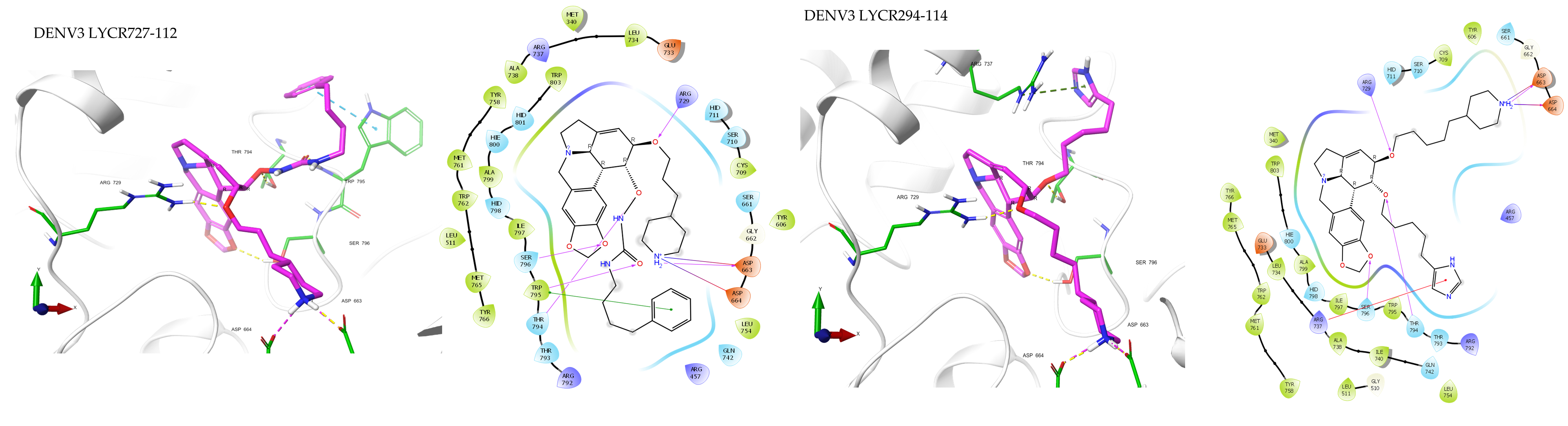

Supplement: Supplementary file 1 [file metabolites-14-00519-s001.zip › Figure S6.png]

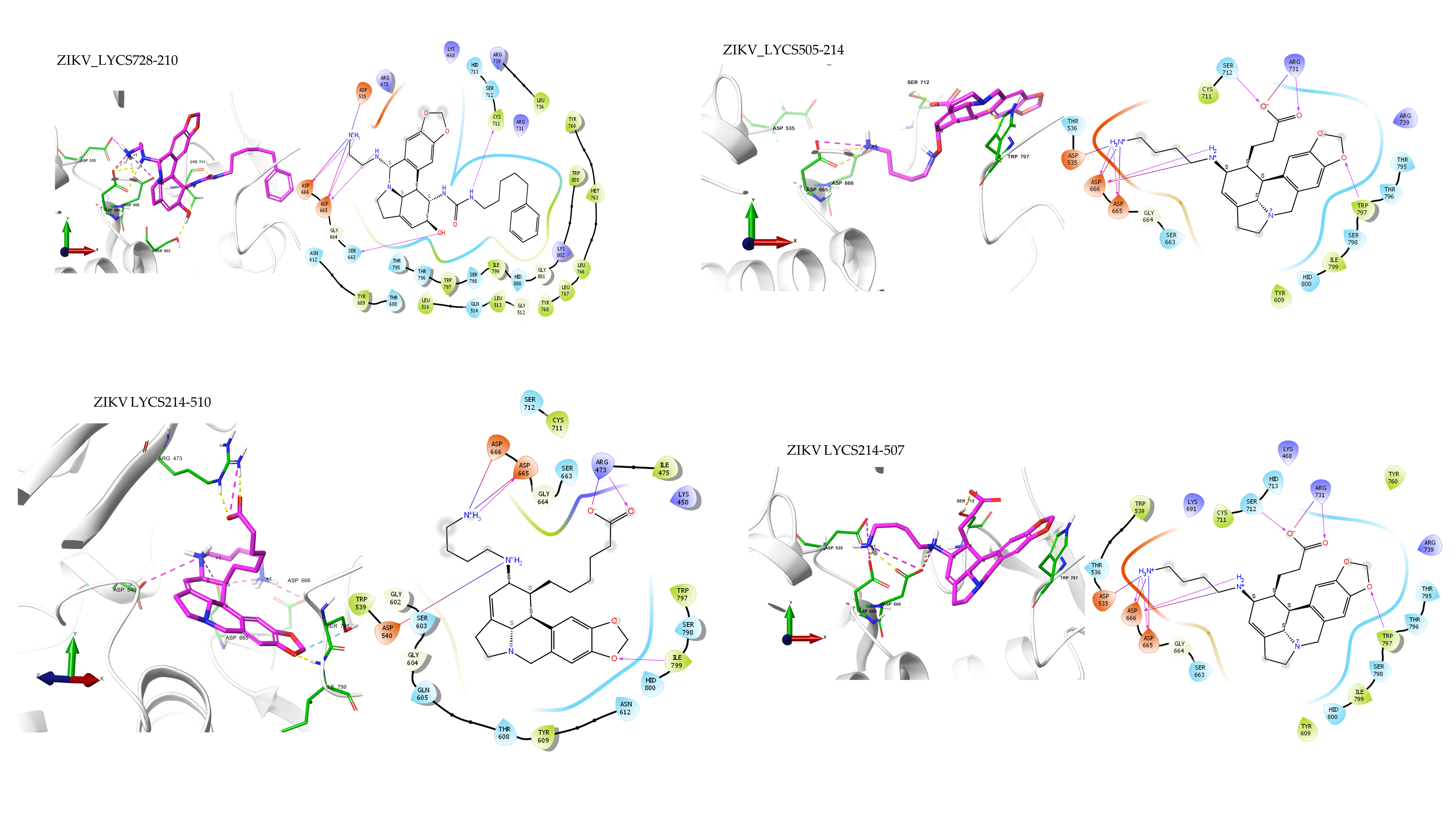

Supplement: Supplementary file 1 [file metabolites-14-00519-s001.zip › Figure S7.png]

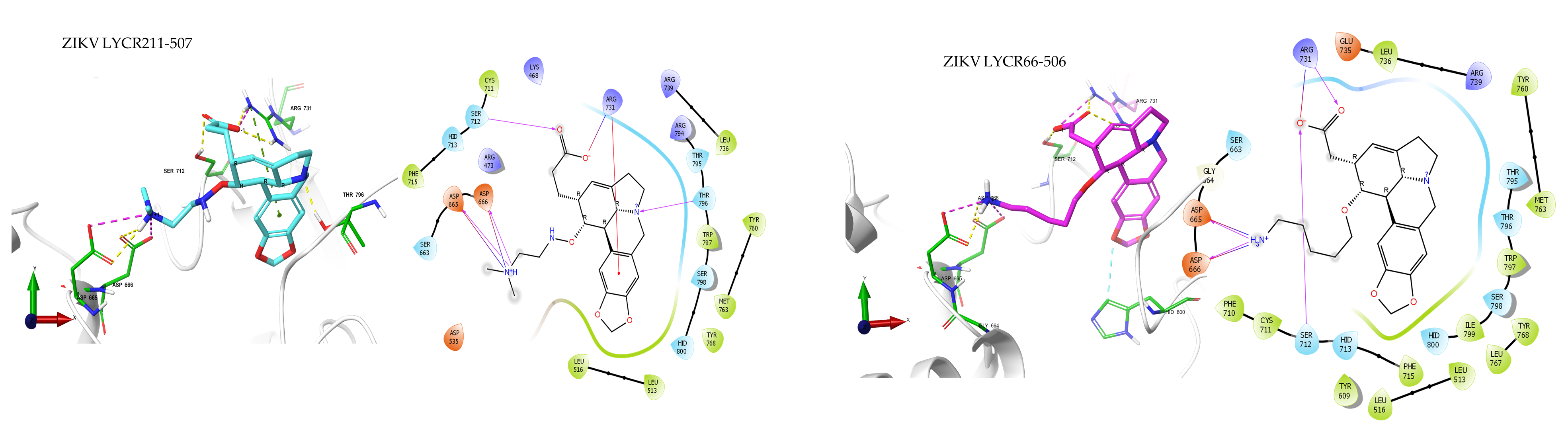

Supplement: Supplementary file 1 [file metabolites-14-00519-s001.zip › Figure S8.png]

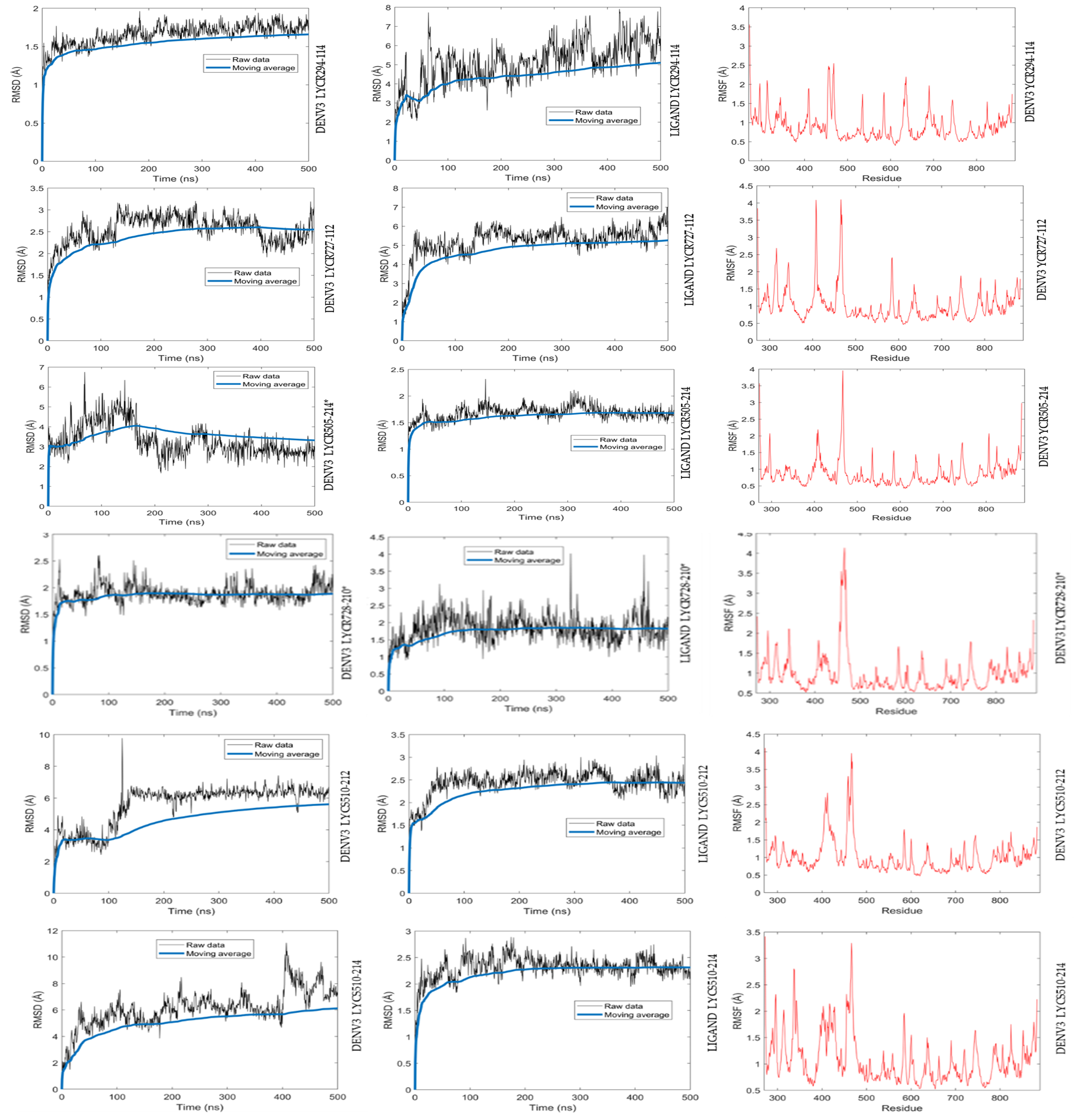

Supplement: Supplementary file 1 [file metabolites-14-00519-s001.zip › Figure S9.png]

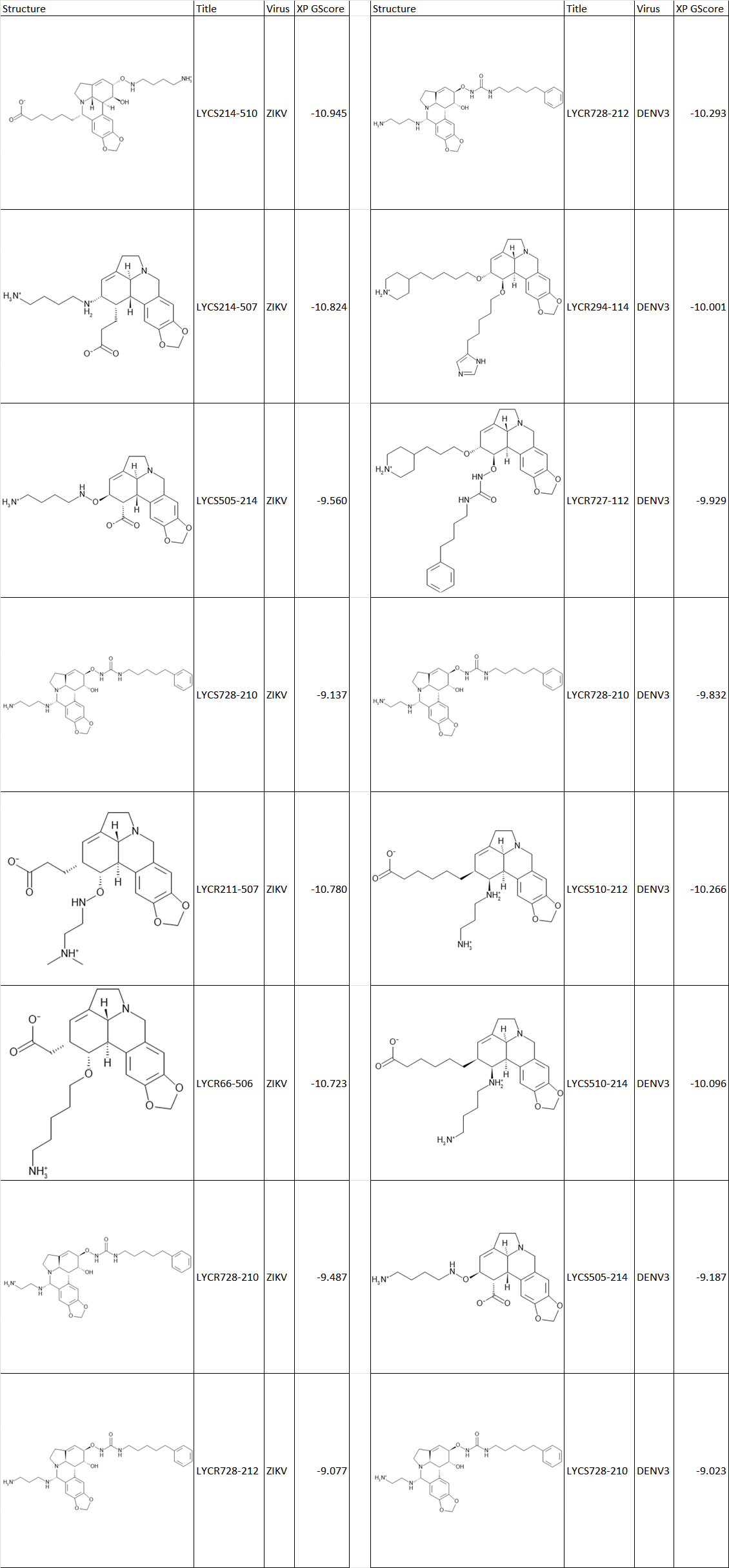

Supplement: Supplementary file 1 [file metabolites-14-00519-s001.zip › Table S1.png]

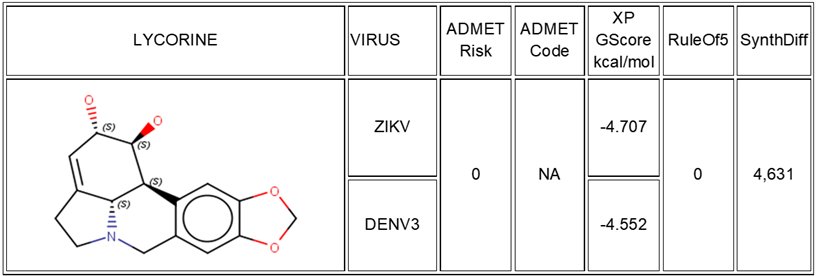

Supplement: Supplementary file 1 [file metabolites-14-00519-s001.zip › Table S2.png]
